# Isolation and characterization of neural stem/progenitor cells in the subventricular zone of the naked mole-rat brain

**DOI:** 10.1186/s41232-021-00182-7

**Published:** 2021-11-01

**Authors:** Yuki Yamamura, Yoshimi Kawamura, Yuki Oiwa, Kaori Oka, Nobuyuki Onishi, Hideyuki Saya, Kyoko Miura

**Affiliations:** 1 Department of Aging and Longevity Research, Faculty of Life Sciences, Kumamoto University, Kumamoto, 860-0811 Japan; 2 Division of Gene Regulation, Institute for Advanced Medical Research, Keio University School of Medicine, Tokyo, 160-0016 Japan; 3 Center for Metabolic Regulation of Healthy Aging, Kumamoto University, Kumamoto, 860-8556 Japan

**Keywords:** Naked mole-rat, Neural stem cell, Cell cycle, Cell proliferation, DNA damage response

## Abstract

**Background:**

The naked mole-rat (NMR) is the longest-lived rodent with a maximum lifespan of more than 37 years and shows a negligible senescence phenotype, suggesting that tissue stem cells of NMRs are highly capable of maintaining homeostasis. However, the properties of NMR tissue stem cells, including neural stem cells (NSCs), are largely unclear.

**Methods:**

Neural stem/progenitor cells (NS/PCs) were isolated from the subventricular zone of the neonate NMR brain (NMR-NS/PCs) and cultured in neurosphere and adherent culture conditions. Expression of NSC markers and markers of neurons, astrocytes, and oligodendrocytes was analyzed by immunocytochemistry. In adherent culture conditions, the proliferation rate and cell cycle of NMR-NS/PCs were assessed and compared with those of NS/PCs from mice (mouse-NS/PCs). The DNA damage response to γ-irradiation was analyzed by immunocytochemistry and reverse transcription-quantitative PCR.

**Results:**

NMR-NS/PCs expressed several NSC markers and differentiated into neurons, astrocytes, and oligodendrocytes. NMR-NS/PCs proliferated markedly slower than mouse-NS/PCs, and a higher percentage of NMR-NS/PCs than mouse-NS/PCs was in G0/G1 phase. Notably, upon γ-irradiation, NMR-NS/PCs exhibited a faster initiation of the DNA damage response and were less prone to dying than mouse-NS/PCs.

**Conclusions:**

NMR-NS/PCs were successfully isolated and cultured. The slow proliferation of NMR-NS/PCs and their resistance to DNA damage may help to prevent stem cell exhaustion in the brain during the long lifespan of NMRs. Our findings provide novel insights into the mechanism underlying delayed aging of NMRs. Further analysis of NMR tissue stem cells may lead to the development of new strategies that can prevent aging in humans.

**Supplementary Information:**

The online version contains supplementary material available at 10.1186/s41232-021-00182-7.

## Background

Naked mole-rats (NMRs) are the longest-lived rodent species and live underground in northeastern Africa [1]. The maximum lifespan of NMRs exceeds 37 years despite their body size being similar to that of laboratory mice [2]. Notably, Ruby et al. reported that NMRs do not show an increase in mortality with age, an important indicator of organismal aging [3]. Moreover, NMRs exhibit extraordinary resistance to cancer; spontaneous carcinogenesis was seldom observed in more than 2000 necropsies of captive NMR colonies [4–6]. These unusual characteristics make NMRs an attractive animal model for obtaining clues to prevent aging and cancer in humans.

Recently, several studies proposed potential mechanisms underlying the longevity and cancer resistance of NMRs involving protein stability [7], activity of nuclear factor, erythroid 2-like 2 (NRF2) signaling [8], translational fidelity [9], production of high molecular mass hyaluronic acid [10], the unique cellular response to reprogramming/oncogenic stress [11, 12], and the retrotransposon [13]. NMRs also have a high DNA repair capacity. Tian et al. reported that compared with short-lived rodent species, fibroblasts from various long-lived rodent species including NMRs have a high DNA double-strand break (DSB) repair capacity and high activity of Sirtuin 6, which acts as a DSB sensor [14, 15]. In addition to their high DSB repair capacity, NMRs have high DNA excision repair activity [16].

Stem cells can self-renew in an undifferentiated state and differentiate into at least one specialized cell type. We and other groups previously generated induced pluripotent stem (iPS) cells from NMR fibroblasts and showed that these cells exhibit marked tumor resistance when transplanted into immunodeficient mice [11, 12, 17]. Tissue stem cells, which exist in various locations such as the bone marrow, intestine, muscle, and brain, play important roles in maintaining body homeostasis and repairing tissues [18]. Depletion or dysfunction of tissue stem cells is one of the major causes of aging and cancer [19]. Neural stem cells (NSCs) are tissue stem cells located in the central nervous system and give rise to neurons, astrocytes, and oligodendrocytes [20]. In the postnatal and adult rodent brain, most NSCs are located in the subventricular zone (SVZ) and the subgranular zone (SGZ) [21]. In these niches, most NSCs are maintained in a quiescent state, which is a reversible state of cell cycle arrest, and a small population of NSCs is activated by various stimuli and generates neurons [22]. The balance between stem cell quiescence and activation is crucial for long-term maintenance of the stem cell pool and the neurogenic capacity of the brain during aging [23]. In addition, recent reports suggested that highly malignant brain tumors originate from neural stem/progenitor cells (NS/PCs) [24, 25]. Therefore, NSCs in NMRs may be highly capable of maintaining cellular homeostasis, which may be related to resistance to aging and cancer.

Adult neurogenesis occurs in the SVZ and SGZ of the NMR brain [26–28]. However, isolation and culture of NMR NSCs have not been previously reported. Therefore, the properties of these cells are largely unknown. In this study, we isolated NS/PC populations from the SVZ of NMR neonates (NMR-NS/PCs) and characterized their basic properties and DNA repair capacity.

## Methods

### Animals

The Ethics Committees of Kumamoto University approved all procedures (approval no. A30-043 and A2020-042). The procedures were in accordance with the Guide for the Care and Use of Laboratory Animals (United States National Institutes of Health). NMRs were maintained at Kumamoto University. C57BL/6N mice were purchased from Japan SLC, Inc.

### Primary culture of NS/PCs

To obtain NMR-NS/PCs and NS/PCs from mice (mouse-NS/PCs), SVZs of neonatal NMRs and C57BL/6N mice at postnatal days 1–2 were isolated, as previously described [29]. The isolated SVZs were washed with ice-cold phosphate-buffered saline (PBS; Nacalai Tesque) containing 0.6% glucose, 1% penicillin/streptomycin (FUJIFILM Wako), and amphotericin B (FUJIFILM Wako). The SVZs were dissociated in growth medium (described below) containing 1.3 mg/ml trypsin (Sigma-Aldrich), 0.7 mg/ml hyaluronidase (Sigma-Aldrich), and 0.1 mg/ml DNase I (Sigma-Aldrich) at 32°C for 10 min. To stop the enzymatic reaction, an equivalent volume of a trypsin inhibitor solution (growth medium containing 2 mg/ml trypsin inhibitor and 0.1 mg/ml DNase I) was added, and the samples were dissociated by pipetting. Cells from each neonate were collected by centrifugation, washed with growth medium, and seeded in a single well of a 6-well plate in growth medium, which comprised Dulbecco’s modified Eagle medium: Nutrient Mixture F-12 (Sigma-Aldrich) supplemented with 2% B27 Supplement minus Vitamin A (Gibco), 0.5% penicillin/streptomycin, 2 mM L-glutamine (Nacalai Tesque or FUJIFILM Wako), 200 ng/ml heparan sulfate sodium salt from bovine kidney (Sigma-Aldrich), and 20 ng/ml of both basic fibroblast growth factor (bFGF; Pepro Tech EC) and epidermal growth factor (EGF; Pepro Tech EC). Half of the medium was replaced with fresh medium every 5 days. When spheres exceeded 100 μm in diameter, they were passaged using 0.05% trypsin-EDTA and 2 mg/ml trypsin inhibitor containing 0.1 mg/ml DNase I, and plated at a density of 1.5 × 10^6^ cells per plate in 100 mm ultra-low attachment dishes (Corning). For adherent culture, cells were plated at a density of 1.5 × 10^6^ cells per plate in poly-L-ornithine/laminin-coated 100 mm plates. For differentiation, neurospheres were plated onto poly-L-ornithine/fibronectin-coated coverslips in a 48-well plate, and cells in adherent culture conditions were plated at a density of 4 × 10^4^ cells per well onto poly-L-ornithine/fibronectin-coated coverslips in a 48-well plate and allowed to differentiate without growth factors for 10 days.

### Immunofluorescence staining

For BrdU labeling, neonates were injected with 100 μg/g body weight BrdU three times every 2 h and sacrificed 2 h after the last injection. Tissues were fixed in 4% paraformaldehyde (PFA; FUJIFILM Wako) at 4°C overnight, and sections were prepared at a thickness of 50 μm using a vibratome. The sections were blocked with 0.5% TNB blocking reagent (PerkinElmer) in PBS containing 0.3% Triton X-100 (Nacalai Tesque) at room temperature (RT) for 2 h. Subsequently, sections were incubated with antibodies against SRY-box transcription factor 2 (SOX2; ab97959, Abcam; 1:300), nestin (NES; non-commercial [30]; 1:300), musashi RNA-binding protein 1 (MSI1; 14-9896, eBioscience; 1:1000), and BrdU (20-BS17, Fitzgerald; 1:500) at 4°C overnight with shaking. After washing with PBS, the sections were incubated with Alexa Fluor 488 or 555-conjugated anti-rabbit IgG (A21424, Thermo Fisher Scientific; 1:1000) to detect SOX2 and NES labeling, Alexa Fluor 555-conjugated anti-rat IgG (A21434, Thermo Fisher Scientific; 1:1000) to detect MSI1 labeling, or Alexa Fluor 555-conjugated anti-sheep IgG (A11015, Life Technologies; 1:1000) to detect BrdU labeling, and Hoechst 33342 (Sigma-Aldrich; 1:1000) diluted in blocking buffer for 2 h at RT, washed with PBS, and mounted in ProLong Glass Antifade Mountant (Thermo Fisher Scientific). Images were captured using a BZ-X 710 or 800 fluorescence microscope (Keyence).

For immunofluorescence staining of cultured cells, cells were fixed with 4% PFA at RT for 10 min. Cells were blocked with 0.5% TNB blocking reagent in PBS with or without 0.3% Triton X-100 at RT. Cells were incubated with primary antibodies against SOX2 (1:300), NES (1:1000), MSI1 (1:1000), tubulin beta 3 class III (TUBB3; ab78078, Abcam; 1:1000), microtubule-associated protein 2 (MAP2; M4403, Sigma-Aldrich; 1:1000), glial fibrillary acidic protein (GFAP; ab7260, Abcam; 1:500), S100 calcium-binding protein B (S100B; ab52642, Abcam; 1:200), oligodendrocyte-specific protein (OSP; ab53041, Abcam; 1:200), O4 (MAB1326, R&D Systems; 1:1000), phosphorylated histone H2AX (γH2AX; 05-636, Merck; 1:1,000), tumor protein p53-binding protein 1 (53BP1; NB100-304, Novus Biologicals; 1:1000), and phosphorylated ATM serine/threonine (pATM; 4526, Cell Signaling Technology; 1:1000). Immunoreactivity was visualized using secondary antibodies conjugated with Alexa 488 or Alexa 555 (Thermo Fisher Scientific). Nuclei were counterstained with Hoechst 33342. Images were captured using a BZ-X 710 or 800 fluorescence microscope. To quantify the percentages of cells positive for each cell type-specific marker, images of four randomly selected microscope fields (at least 100 cells) per cell line were captured, and the numbers of positive cells were manually counted. To quantify γH2AX, 53BP1, and pATM foci, images of four randomly selected microscope fields (at least 100 cells) per cell line were captured, and the average intensity of γH2AX per nuclei was measured by averaging the fluorescence intensity of γH2AX in the nucleus using a BZ-X Image Analyzer (Keyence), and cells with >10 53BP1 and pATM foci were manually counted.

### Cell growth analysis

NMR- and mouse-NS/PCs were seeded at a density of 8 × 10^4^ cells per well in a 24-well plate. Seeded cells were trypsinized and counted every 3 days using a Coulter Counter (Beckman Coulter). The population doubling time was estimated from the slope of the growth curve.

### Cell cycle analysis

Cells in the logarithmic growth phase were collected and fixed with 70% ethanol at 4°C for 2 h. After two washes with PBS, cells were incubated with RNase A (QIAGEN, final concentration of 0.25 mg/ml) at 37°C for 45 min and then stained with propidium iodide (PI; FUJIFILM Wako, final concentration of 50 μg/ml) at 4°C for 30 min in the dark. Flow cytometry was performed using a FACSVerse flow cytometer (BD Biosciences). Data were analyzed using FlowJo 10 software (BD Biosciences).

### γ-Irradiation and cell death analysis

Cells were seeded into 24-well plates at a density of 1.6 × 10^5^ cells per well. At 24 h after seeding, cells were exposed to 10 Gy of γ-radiation. To detect cell death, cells were stained with Hoechst 33342 (Sigma; 1 mg/ml; 1:1000 in growth medium) for 10 min at 32°C and then stained with PI (10 mg/ml; 1:1000 in growth medium) for 10 min at 32°C. Images were captured using a BZ-X 710 fluorescence microscope (Keyence) and positively stained cells were counted using a BZ-X Image Analyzer (Keyence). All cells in 12 randomly selected microscope fields (at least 10,000 cells) per cell line were analyzed for PI- and Hoechst-positive nuclei. PI- and Hoechst-double-positive cells were counted as dead cells.

### Reverse transcription-quantitative PCR (RT-qPCR)

Total RNA was extracted using TRIzol (Thermo Fisher Scientific) according to the manufacturer’s protocol. Genomic DNA was removed using gDNA Eliminator spin columns (Qiagen). RNA was eluted from the columns using 30 μl of RNase-free water and quantified using a NanoDrop spectrophotometer (Thermo Scientific). cDNA was synthesized with ReverTra Ace qPCR RT Master Mix (TOYOBO) using 400 ng of total RNA input. RT-qPCR assays were set up in triplicate using PowerUp SYBR Green Master Mix (Invitrogen) and run on a CFX384 Touch Real-Time PCR Detection System (Bio-Rad). Primer sequences are listed in Supplementary Table 1.

### Statistical analysis

GraphPad Prism was used for statistical analysis. Data are presented as the mean ± standard deviation (SD). Data were analyzed using a two-way analysis of variance (ANOVA) followed by Bonferroni’s multiple comparisons test or a one-way ANOVA followed by Dunnett’s multiple comparison test. The unpaired *t*-test was used to compare two groups.

## Results

### Isolation and characterization of NMR-NS/PCs

**Fig. 1:**
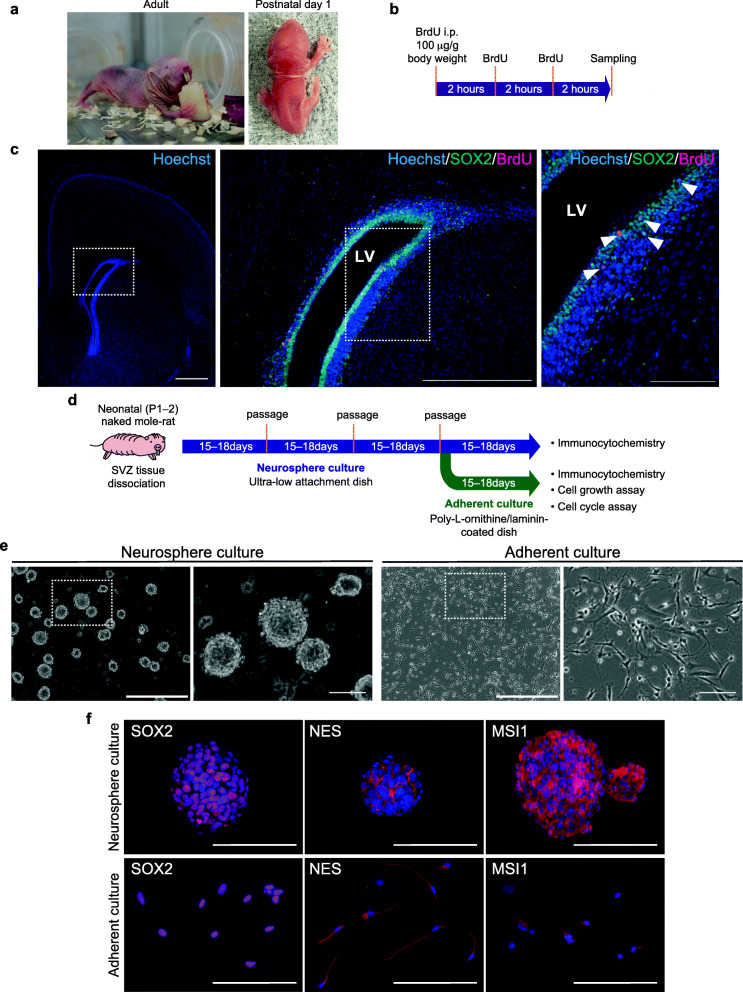
Isolation and characterization of neural stem/progenitor cells (NS/PCs) from the subventricular zone (SVZ) of neonatal naked mole-rats (NMRs). **a** An adult NMR (left) and a NMR at postnatal day 1 (right). **b** Timeline of the experimental procedure for BrdU labeling. **c** Immunofluorescence images of SRY-box transcription factor 2 (SOX2; green), BrdU (magenta), and Hoechst 33342 (blue) in the neonatal NMR SVZ. Left, a low magnification image of Hoechst staining in the NMR neonatal brain. Center, an enlarged image of the boxed region in the left image. Right, a higher magnification image of the boxed region in the center image. White arrowheads indicate SOX2/BrdU double-positive cells. Scale bars: 500 μm (left), 500 μm (center), and 100 μm (right). LV, lateral ventricle. **d** Timeline of the experimental procedure for primary neurosphere and adherent culture of NS/PCs from the NMR SVZ. **e** Morphologies of NMR-NS/PCs under neurosphere and adherent culture conditions. The boxed regions in the left images are enlarged in the right images. Scale bars: 500 μm (left) and 100 μm (right). **f** Immunofluorescence images of SOX2, nestin (NES), and musashi RNA-binding protein 1 (MSI1) in NMR-NS/PCs under each culture condition. Blue, Hoechst 33342. Scale bars: 100 μm

We isolated NS/PCs from early neonatal (postnatal days 1–2, P1–2) NMRs (Fig. 1a). Immunohistochemistry showed that the SVZ of the neonatal NMR brain contained many cells positive for the NSC markers SOX2, NES, and MSI1, as well as a small population of BrdU (a proliferation marker)-positive cells (Fig. 1b, c, Supplementary Fig. 1a). In the NMR SVZ, there were many BrdU-positive cells not only in the lateral wall but also in the medial wall (Supplementary Fig. 1b). Upon immunohistochemical staining of the SVZ for SOX2, strongly positive cells were observed in the area near the wall and weakly positive cells were observed in the area distant from the wall (Supplementary Fig. 1c). Although the amino acid sequence of NES varies among species, the amino acid homology of the antigen site between the human and NMR proteins is 73.3%, and the antibody showed cross-reactivity (Supplementary Fig. 1d). To isolate and culture NS/PCs, the SVZ of NMRs was microdissected as previously described in mice (Fig. 1d) [29]. The dissected SVZ was dissociated by gentle pipetting after treatment with trypsin and hyaluronidase, suspended in NSC culture medium supplemented with B27, bFGF, and EGF, as previously used in other species [31], and cultured in neurosphere culture conditions. The NMR is a unique heterothermic animal with a low body temperature and its cells are vulnerable to culture in normoxia [17, 32]; therefore, the cells were cultured at 32°C and in 3% O_2_. Neurospheres formed after 15 days of cultivation (Fig. 1e). To eliminate adherent differentiated cells, neurospheres were cultured in a cell culture flask and passaged three times. Dissociated neurospheres could grow on poly-L-ornithine/laminin-coated dishes (adherent culture, Fig. 1e). These cells could be cultured up to at least passage six in both neurosphere and adherent culture conditions. Immunocytochemistry showed that most cells were positive for the NSC markers SOX2, NES, and MSI1 in both culture conditions (Fig. 1f, Supplementary Fig. 2a). These results indicate that NMR-NS/PCs can proliferate while remaining in an undifferentiated state using the neurosphere or adherent culture protocol.

### NMR-NS/PCs proliferate slower than mouse-NS/PCs

**Fig. 2:**
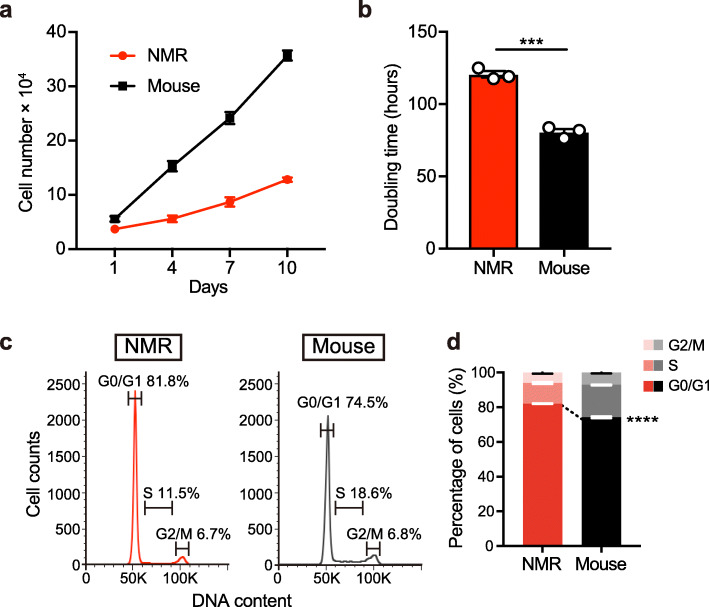
Comparison of the proliferative potentials of neural stem/progenitor cells from naked mole-rats (NMR-NS/PCs) and mice (mouse-NS/PCs). **a** Growth of NMR- and mouse-NS/PCs in adherent culture conditions. Data are mean ± standard deviation (SD) of *n* = 3 biological triplicates. **b** Doubling time of NMR- and mouse-NS/PCs in adherent culture conditions. Data are mean ± SD of *n* = 3 biological triplicates. ***P < 0.001, unpaired t-test. **c** Representative cell cycle profile plot based on the DNA content determined by flow cytometry for NMR- and mouse-NS/PCs. **d** Quantification of the percentages of NMR- and mouse-NS/PCs in each cell cycle phase. Data are mean ± SD of *n* = 3 biological triplicates. ****P < 0.0001, unpaired *t*-test

Proliferation of NMR-NS/PCs in adherent culture was compared with that of mouse-NS/PCs under the same temperature and oxygen conditions (32°C, 3% O_2_). NMR-NS/PCs proliferated slower than mouse-NS/PCs (Fig. 2a). The doubling time of NMR-NS/PCs was 120 h, which was 1.5 times longer than that of mouse-NS/PCs (80 h) (Fig. 2b). Cell cycle analysis by flow cytometry showed that a higher percentage of NMR-NS/PCs than mouse-NS/PCs was in G0/G1 phase, suggesting that NMR-NS/PCs have a longer G1 phase or more NMR-NS/PCs are in G0 phase (Fig. 2c, d). NMR-NS/PCs could not proliferate at 37°C and in 21% O_2_, the standard culture condition for mouse-NS/PCs (Supplementary Fig. 2b). These results indicate that NMR-NS/PCs have a lower proliferation potential than mouse-NS/PCs.

### NMR-NS/PCs can differentiate into three neural lineages

**Fig. 3:**
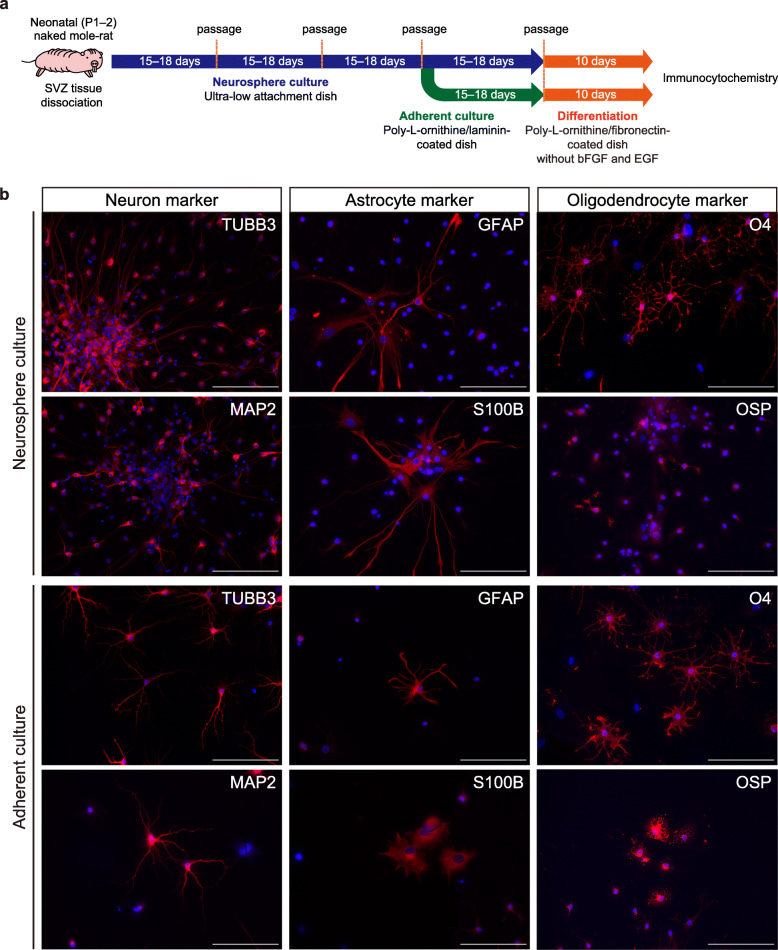
Differentiation potential of neural stem/progenitor cells from naked mole-rats (NMR-NS/PCs). **a** Timeline of the experimental procedure to induce differentiation of NMR-NS/PCs. **b** Immunofluorescence images of differentiated NMR-NS/PCs under neurosphere (upper panels) and adherent (lower panels) culture conditions. Cells were labeled for neuron markers [tubulin beta 3 class III (TUBB3) and microtubule-associated protein 2 (MAP2)], astrocyte markers [glial fibrillary acidic protein (GFAP) and S100 calcium-binding protein B (S100B)], and oligodendrocyte markers [O4 and oligodendrocyte-specific protein (OSP)]. Blue, Hoechst 33342. Scale bars: 100 μm

To induce differentiation, NMR-NS/PCs grown in neurosphere or adherent culture conditions were plated onto poly-L-ornithine/fibronectin-coated plates and cultured for 10 days without bFGF and EGF as previously described in mice (Fig. 3a) [33]. Immunocytochemistry showed that differentiated cells were positive for neuron markers (TUBB3 and MAP2), astrocyte markers (GFAP and S100B), and oligodendrocyte markers (O4 and OSP) (Fig. 3b, Supplementary Fig. 2c). These data suggest the possibility that cultured NMR-NS/PCs can differentiate into three neural lineages.

### NMR-NS/PCs are resistant to DNA damage induced by γ-irradiation

**Fig. 4:**
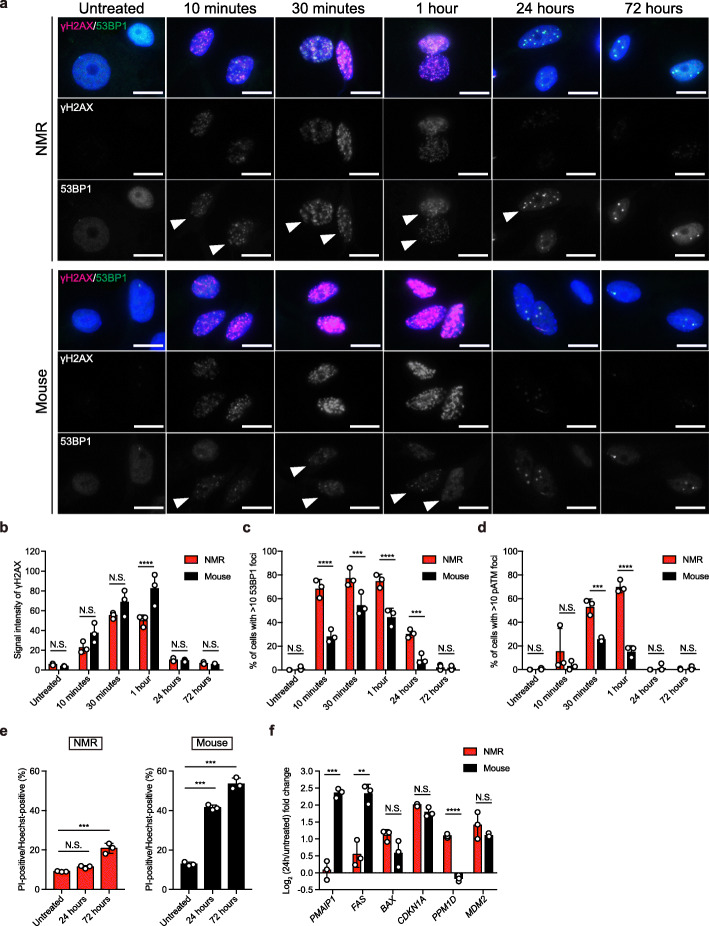
Comparison of the DNA damage response after γ-irradiation between neural stem/progenitor cells from naked mole-rats (NMR-NS/PCs) and mice (mouse-NS/PCs). **a** Immunofluorescence images of phosphorylated histone H2AX (γH2AX; magenta) and tumor protein p53-binding protein 1 (53BP1; green) in NMR- and mouse-NS/PCs exposed to 10 Gy of γ-radiation or left untreated. Blue, Hoechst 33342. White arrowheads indicate positive cells with >10 53BP1 foci per nuclei. Scale bars: 10 μm. **b**–**d** Quantification of the average signal intensity of γH2AX per nuclei (**b**), cells with >10 53BP1 foci (**c**), and cells with >10 phosphorylated ATM serine/threonine (pATM) foci (**d**). Data are mean ± standard deviation (SD) from *n* = 3 biological replicates. ***P < 0.001; ****P < 0.0001; N.S., not significant, two-way analysis of variance (ANOVA) followed by Bonferroni’s multiple comparisons test. **e** Quantification of cell death using propidium iodide (PI)/Hoechst staining after γ-irradiation. Data are mean ± SD from *n* = 3 biological replicates. ***P < 0.001; N.S., not significant, one-way ANOVA followed by Dunnett’s multiple comparison test. **f** Relative expression of genes downstream of p53 at 24 h after γ-irradiation determined by reverse transcription-quantitative PCR. Fold changes were calculated relative to the untreated control. Data are mean ± SD from *n* = 3 biological replicates. **P < 0.01; ***P < 0.001; ****P < 0.0001; N.S., not significant, unpaired *t*-test

To evaluate the response of NMR-NS/PCs to DNA damage, we performed γ-irradiation (Supplementary Fig. 3a). Immunocytochemistry and quantification of γH2AX, a DSB marker [34], showed that the signal intensity of γH2AX at 1 h after γ-irradiation was significantly lower in NMR-NS/PCs than in mouse-NS/PCs, suggesting that DNA damage is less severe in NMR-NS/PCs than in mouse-NS/PCs (Fig. 4a, b). We then examined changes in the foci numbers of 53BP1 and pATM, which are DNA damage response markers, in nuclei after γ-irradiation [35, 36]. Notably, more 53BP1 foci had formed in NMR-NS/PCs than in mouse-NS/PCs at 10 min after γ-irradiation, and the number of 53BP1 foci remained high in NMR-NS/PCs until 24 h (Fig. 4a, c). γH2AX and 53BP1 tended to be colocalized more frequently in NMR-NS/PCs than in mouse-NS/PCs at 10 min, 30 min, and 1 h after γ-irradiation (Fig. 4a). Similarly, more pATM foci had formed in NMR-NS/PCs than in mouse-NS/PCs at 30 min and 1 h after γ-irradiation (Fig. 4d, Supplementary Fig. 3b). These results suggest that DNA repair is initiated faster in NMR-NS/PCs than in mouse-NS/PCs. After 72 h, γH2AX signals decreased in both NMR- and mouse-NS/PCs, and 53BP1 and pATM foci had returned to the levels observed before γ-irradiation (Fig. 4b–d). Consistent with the data suggesting a fast DNA damage response, NMR-NS/PCs were less prone to dying upon γ-irradiation than mouse-NS/PCs. At 24 h after exposure to 10 Gy of γ-radiation, the percentage of dead NMR-NS/PCs was not significantly increased (Fig. 4e). At 72 h after exposure to 10 Gy of γ-radiation, about 20% of NMR-NS/PCs were dead, in contrast with about 50% of mouse-NS/PCs (Fig. 4e). p53 responds to DNA damage and induces expression of genes involved in both cell cycle arrest and apoptosis [37, 38]. At 24 h after γ-radiation, RT-qPCR analysis of genes downstream of p53 was performed. Among cell death-related genes, expression of BCL2-associated X, apoptosis regulator *(BAX)* was upregulated in both NMR- and mouse-NS/PCs upon γ-irradiation, whereas expression of phorbol-12-myristate-13-acetate-induced protein 1 (*PMAIP1*, also known as *NOXA*) and Fas cell surface death receptor *(FAS)* was upregulated less in NMR-NS/PCs than in mouse-NS/PCs. In terms of cell cycle arrest-related genes, cyclin-dependent kinase inhibitor 1A (*CDKN1A*), protein phosphatase, Mg^2+^/Mn^2+^-dependent 1D (*PPM1D*, also known as *WIP1*), and MDM2 proto-oncogene *(MDM2)* were upregulated in NMR-NS/PCs (Fig. 4f). These differences in expression of genes downstream of p53 are consistent with fewer NMR-NS/PCs dying upon γ-irradiation. Taken together, our results indicate that NMR-NS/PCs are more resistant to γ-irradiation than mouse-NS/PCs.

## Discussion

In this study, we successfully isolated and cultured NS/PCs from the SVZ of neonatal NMRs. Compared with mouse-NS/PCs, NMR-NS/PCs proliferated significantly slower and more cells were in G0/G1 phase. Notably, NMR-NS/PCs were more resistant to γ-irradiation than mouse-NS/PCs.

In this study, we compared the characteristics of NS/PCs derived from the SVZs of NMRs and mice at postnatal days 1–2. A previous study reported that the numbers of SOX2-positive cells in the SVZs of mouse and NMR neonates are similar [39]. On the other hand, Orr et al. showed that the brains of postnatal NMRs are more developed than those of mice and have a clearly laminated hippocampus and myelinated white matter tracts, similar to brains of neonatal primates. On the other hand, postnatal NMR brains mature slower than mouse brains. Thus, the developmental stages of NMR SVZs and the properties of NSCs derived from them must be compared with those of mice in more detail in the future, not only in the neonatal stage but also in the fetal stage.

Proliferation of NMR skin fibroblasts is reportedly suppressed by induction of early contact inhibition via INK4A activation due to signaling of high molecular mass hyaluronan [10, 40]. On the other hand, we previously showed that NMR iPS cells have a lower proliferative capacity than mouse iPS cells [11]. In this study, we revealed that proliferation of NMR-NS/PCs is lower than that of mouse-NS/PCs. These two types of NMR stem cells do not exhibit an early contact inhibition phenotype, suggesting that cell cycle progression is negatively regulated in several types of NMR cells. Further studies of cell cycle control in NMR cells are required to better understand longevity and cancer resistance in this species.

Genome instability is closely related to aging and cancer. It has been suggested that long-lived mammals have a high DNA repair capacity [41]. In this study, we found that the DNA damage response of NMR-NS/PCs has unique characteristics. In the early stage, NMR-NS/PCs showed less DNA damage (signal intensity of γH2AX) and more repair sites (numbers of 53BP1 and pATM foci) than mouse-NS/PCs. Previous reports suggested that some DNA repair pathways are highly efficient in NMRs due to high poly ADP-ribosylation activity [14, 16, 42]. Poly ADP-ribosylation is an essential process in the early stages of the DNA damage response [43]. Therefore, high poly ADP-ribosylation activity may contribute to efficient DNA repair in NMR-NS/PCs. In the late stage, irradiated NMR-NS/PCs were resistant to death. NMR fibroblasts are less prone to undergo acute death than mouse fibroblasts upon γ-irradiation [44]. The low ability to induce death of NMR-NS/PCs upon DNA damage may contribute to the long-term maintenance of the stem cell pool during the long lifespan of NMRs.

In contrast with NMR cells, lymphocytes from cancer-resistant elephants are highly vulnerable to γ-irradiation and prone to death [45]. This is thought to be one of the mechanisms underlying cancer resistance in elephants via efficiently eliminating mutant cells with damaged DNA from tissues. Hence, the low ability to induce death of NMR cells after induction of DNA damage by γ-irradiation might be a double-edged sword because it may leave mutant cells with damaged DNA in the tissues of NMRs. Therefore, NMR cells may have a very accurate DNA repair system that suppresses the appearance of mutant cells without killing damaged cells. Further analyses, such as comet assays, karyotyping, or genomic sequencing, are required to determine whether accurate DNA repair occurs in NMR cells that survive after DNA damage. It is also necessary to analyze whether poly ADP-ribosylation affects efficient DNA repair in NMR-NS/PC as well as in NMR-fibroblasts [14, 16, 42].

## Conclusions

In this study, we successfully isolated and cultured NS/PCs from the SVZ of neonatal NMRs. The slow proliferation and fast DNA damage response of NMR-NS/PCs may contribute to the delayed-aging phenotype in brain tissues of NMRs by preventing stem cell depletion and maintaining the stem cell pool. Future research of NMR somatic stem cells may lead to the elucidation of novel mechanisms that prevent stem cell exhaustion and dysfunction during aging.

## Supplementary Information

**Additional file 1: Supplementary Table 1.** List of primers used for reverse transcription-quantitative PCR.

**Additional file 2: Supplementary Figure 1.** Immunohistochemical analysis of the neonatal naked mole-rat (NMR) subventricular zone (SVZ). a Immunofluorescence images of nestin (NES) and musashi RNA-binding protein 1 (MSI1) in the neonatal NMR SVZ. The inset shows an overview of Hoechst staining of a coronal hemisection of the neonatal NMR brain. The boxed region in the inset was enlarged. Scale bars: 100 μm (main image) and 500 μm (inset). LV, lateral ventricle. b Immunofluorescence images of BrdU (red) and Hoechst 33342 (blue) in the neonatal NMR SVZ. The boxed regions in the left image are enlarged in the center and right images. Scale bars: 500 μm (left), 100 μm (center), and 100 μm (right). LV, lateral ventricle. c Immunofluorescence image of SRY-box transcription factor 2 (SOX2) in the neonatal NMR SVZ. Scale bar: 100 μm. LV, lateral ventricle. d Multiple alignment of the antigen sites recognized by anti-NES antibodies in the human, NMR, and mouse proteins.

**Additional file 3: Supplementary Figure 2.** Quantification of the percentages of cells positive for each cell type-specific marker and comparison of the growth of neural stem/progenitor cells (NS/PCs) from naked mole-rats (NMR) and mouse in standard culture condition for mouse-NS/PCs. a Proportions of cells positive for each neural stem cell marker in adherent culture conditions. Data are mean ± standard deviation (SD) from *n* = 3 biological replicates. b Growth of NMR-NS/PCs and mouse-NS/PCs in adherent culture conditions at 37°C and in 21% O_2_. Data are mean ± SD of *n* = 3 biological triplicates. c Proportions of cells positive for each cell type-specific marker in adherent culture conditions. Data are mean ± SD from *n* = 3 biological replicates.

**Additional file 4: Supplementary Figure 3.** Analysis of the DNA damage response after γ-irradiation. a Timeline of the experimental procedure for γ-irradiation. b Immunofluorescence images of phosphorylated ATM serine/threonine (pATM) in naked mole-rat (NMR) and mouse neural stem/progenitor cells exposed to γ-radiation or left untreated. Blue, Hoechst 33342. White arrowheads indicate positive cells with >10 pATM foci per nuclei. Scale bars: 10 μm.

## Data Availability

The datasets used and/or analyzed during the current study are available from the corresponding author on reasonable request.

## Abbreviations

NMR: Naked mole-rat
NSC: Neural stem cell
NS/PC: Neural stem/progenitor cell
NRF2: Nuclear factor, erythroid 2-like 2
DSB: DNA double-strand break
iPS: Induced pluripotent stem
SVZ: Subventricular zone
SGZ: Subgranular zone
LV: Lateral ventricle
PBS: Phosphate-buffered saline
bFGF: Basic fibroblast growth factor
EGF: Epidermal growth factor
PFA: Paraformaldehyde
RT: Room temperature
SOX2: SRY-box transcription factor 2
NES: Nestin
MSI1: Musashi RNA-binding protein 1
TUBB3: Tubulin beta 3 class III
MAP 2: Microtubule-associated protein 2
GFAP: Glial fibrillary acidic protein
S100B: S100 calcium-binding protein B
OSP: Oligodendrocyte-specific protein
γH2AX: Phosphorylated histone H2AX
53BP1: Tumor protein p53-binding protein 1
pATM: Phosphorylated ATM serine/threonine
PI: Propidium iodide
SD: Standard deviation
ANOVA: Analysis of variance
P: Postnatal
RT-qPCR: Reverse transcription-quantitative PCR
BAX: BCL2-associated X, apoptosis regulator
PMAIP1: Phorbol-12-myristate-13-acetate-induced protein 1
FAS: Fas cell surface death receptor
CDKN1A: Cyclin-dependent kinase inhibitor 1A
PPM1D: Protein phosphatase, Mg^2+^/Mn^2+^-dependent 1D
MDM2: MDM2 proto-oncogene

## References

[1] Jarvis JUM (1981). Eusociality in a mammal: cooperative breeding in naked mole-rat colonies. Science..

[2] Lee BP, Smith M, Buffenstein R, Harries LW (2020). Negligible senescence in naked mole rats may be a consequence of well-maintained splicing regulation. GeroScience..

[3] Ruby JG, Smith M, Buffenstein R (2018). Naked mole-rat mortality rates defy gompertzian laws by not increasing with age. Elife..

[4] Buffenstein R (2008). Negligible senescence in the longest living rodent, the naked mole-rat: Insights from a successfully aging species. J Comp Physiol B..

[5] Delaney MA, Ward JM, Walsh TF, Chinnadurai SK, Kerns K, Kinsel MJ, Treuting PM (2016). Initial case reports of cancer in naked mole-rats (Heterocephalus glaber). Vet Pathol..

[6] Taylor KR, Milone NA, Rodriguez CE (2017). Four cases of spontaneous neoplasia in the naked mole-rat (Heterocephalus glaber), a putative cancer-resistant species. J Gerontol A Biol Sci Med Sci..

[7] Pérez VI, Buffenstein R, Masamsetti V, Leonard S, Salmon AB, Mele J (2009). Protein stability and resistance to oxidative stress are determinants of longevity in the longest-living rodent, the naked mole-rat. Proc Natl Acad Sci U S A..

[8] Lewis KN, Wason E, Edrey YH, Kristan DM, Nevo E, Buffenstein R (2015). Regulation of Nrf2 signaling and longevity in naturally long-lived rodents. Proc Natl Acad Sci..

[9] Azpurua J, Ke Z, Chen IX, Zhang Q, Ermolenko DN, Zhang ZD, Gorbunova V, Seluanov A (2013). Naked mole-rat has increased translational fidelity compared with the mouse, as well as a unique 28S ribosomal RNA cleavage. Proc Natl Acad Sci U S A..

[10] Tian X, Azpurua J, Hine C, Vaidya A, Myakishev-Rempel M, Ablaeva J, Mao Z, Nevo E, Gorbunova V, Seluanov A (2013). High-molecular-mass hyaluronan mediates the cancer resistance of the naked mole rat. Nature..

[11] Miyawaki S, Kawamura Y, Oiwa Y, Shimizu A, Hachiya T, Bono H, Koya I, Okada Y, Kimura T, Tsuchiya Y, Suzuki S, Onishi N, Kuzumaki N, Matsuzaki Y, Narita M, Ikeda E, Okanoya K, Seino KI, Saya H, Okano H, Miura K (2016). Tumour resistance in induced pluripotent stem cells derived from naked mole-rats. Nat Commun..

[12] Tan L, Ke Z, Tombline G, Macoretta N, Hayes K, Tian X, Lv R, Ablaeva J, Gilbert M, Bhanu NV, Yuan ZF, Garcia BA, Shi YG, Shi Y, Seluanov A, Gorbunova V (2017). Naked mole rat cells have a stable epigenome that resists iPSC reprogramming. Stem Cell Reports..

[13] Yamaguchi S, Nohara S, Nishikawa Y, Suzuki Y, Kawamura Y, Miura K, Tomonaga K, Ueda K, Honda T (2021). Characterization of an active LINE-1 in the naked mole-rat genome. Sci Rep..

[14] Tian X, Firsanov D, Zhang Z, Cheng Y, Luo L, Tombline G, Tan R, Simon M, Henderson S, Steffan J, Goldfarb A, Tam J, Zheng K, Cornwell A, Johnson A, Yang JN, Mao Z, Manta B, Dang W, Zhang Z, Vijg J, Wolfe A, Moody K, Kennedy BK, Bohmann D, Gladyshev VN, Seluanov A, Gorbunova V (2019). SIRT6 is responsible for more efficient DNA double-strand break repair in long-lived species. Cell..

[15] Onn L, Portillo M, Ilic S, Cleitman G, Stein D, Kaluski S, Shirat I, Slobodnik Z, Einav M, Erdel F, Akabayov B, Toiber D (2020). SIRT6 is a DNA double-strand break sensor. Elife..

[16] Evdokimov A, Kutuzov M, Petruseva I, Lukjanchikova N, Kashina E, Kolova E (2018). Naked mole rat cells display more efficient excision repair than mouse cells. Aging (Albany NY).

[17] Lee SG, Mikhalchenko AE, Yim SH, Lobanov AV, Park JK, Choi KH, Bronson RT, Lee CK, Park TJ, Gladyshev VN (2017). Naked mole rat induced pluripotent stem cells and their contribution to interspecific chimera. Stem Cell Reports..

[18] Ermolaeva M, Neri F, Ori A, Rudolph KL (2018). Cellular and epigenetic drivers of stem cell ageing. Nat Rev Mol Cell Biol..

[19] López-Otín C, Blasco MA, Partridge L, Serrano M, Kroemer G (2013). The hallmarks of aging. Cell..

[20] Okano H, Temple S (2009). Cell types to order: temporal specification of CNS stem cells. Curr Opin Neurobiol..

[21] Bond AM, Ming GL, Song H (2015). Adult mammalian neural stem cells and neurogenesis: five decades later. Cell Stem Cell..

[22] Alvarez-Buylla A, García-Verdugo JM (2002). Neurogenesis in adult subventricular zone. J Neurosci.

[23] Urbán N, Blomfield IM, Guillemot F (2019). Quiescence of adult mammalian neural stem cells: a highly regulated rest. Neuron..

[24] Alcantara Llaguno S, Chen J, Kwon CH, Jackson EL, Li Y, Burns DK, Alvarez-Buylla A, Parada LF (2009). Malignant astrocytomas originate from neural stem/progenitor cells in a somatic tumor suppressor mouse model. Cancer Cell..

[25] Alcantara Llaguno SR, Wang Z, Sun D, Chen J, Xu J, Kim E, Hatanpaa KJ, Raisanen JM, Burns DK, Johnson JE, Parada LF (2015). Adult lineage-restricted CNS progenitors specify distinct glioblastoma subtypes. Cancer Cell..

[26] Amrein I, Becker AS, Engler S, Huang SH, Müller J, Slomianka L (2014). Adult neurogenesis and its anatomical context in the hippocampus of three mole-rat species. Front Neuroanat..

[27] Peragine DE, Simpson JA, Mooney SJ, Lovern MB, Holmes MM (2014). Social regulation of adult neurogenesis in a eusocial mammal. Neuroscience..

[28] Penz OK, Fuzik J, Kurek AB, Romanov R, Larson J, Park TJ, Harkany T, Keimpema E (2015). Protracted brain development in a rodent model of extreme longevity. Sci Rep..

[29] Walker TL, Kempermann G. One mouse, two cultures: isolation and culture of adult neural stem cells from the two neurogenic zones of individual mice. J Vis Exp. 2014;(84):e51225. 10.3791/51225. 10.3791/51225 PMC4131911 24637893

[30] Nakamura Y, Yamamoto M, Oda E, Yamamoto A, Kanemura Y, Hara M, Suzuki A, Yamasaki M, Okano H (2003). Expression of tubulin beta II in neural stem/progenitor cells and radial fibers during human fetal brain development. Lab Invest..

[31] Conti L, Pollard SM, Gorba T, Reitano E, Toselli M, Biella G, Sun Y, Sanzone S, Ying QL, Cattaneo E, Smith A (2005). Niche-independent symmetrical self-renewal of a mammalian tissue stem cell. PLoS Biol..

[32] Oiwa Y, Oka K, Yasui H, Higashikawa K, Bono H, Kawamura Y, Miyawaki S, Watarai A, Kikusui T, Shimizu A, Okano H, Kuge Y, Kimura K, Okamatsu-Ogura Y, Miura K (2020). Characterization of brown adipose tissue thermogenesis in the naked mole-rat (Heterocephalus glaber), a heterothermic mammal. Sci Rep..

[33] Miura K, Okada Y, Aoi T, Okada A, Takahashi K, Okita K, Nakagawa M, Koyanagi M, Tanabe K, Ohnuki M, Ogawa D, Ikeda E, Okano H, Yamanaka S (2009). Variation in the safety of induced pluripotent stem cell lines. Nat Biotechnol..

[34] Rogakou EP, Boon C, Redon C, Bonner WM (1999). Megabase chromatin domains involved in DNA double-strand breaks in vivo. J Cell Biol..

[35] Panier S, Boulton SJ (2014). Double-strand break repair: 53BP1 comes into focus. Nat Rev Mol Cell Biol..

[36] Huang RX, Zhou PK (2020). DNA damage response signaling pathways and targets for radiotherapy sensitization in cancer. Signal Transduct Target Ther..

[37] Roos WP, Thomas AD, Kaina B (2016). DNA damage and the balance between survival and death in cancer biology. Nat Rev Cancer..

[38] Zhang XP, Liu F, Wang W (2011). Two-phase dynamics of p53 in the DNA damage response. Proc Natl Acad Sci U S A..

[39] Orr ME, Garbarino VR, Salinas A, Buffenstein R (2016). Extended postnatal brain development in the longest-lived rodent: prolonged maintenance of neotenous traits in the naked mole-rat brain. Front Neurosci..

[40] Seluanov A, Hine C, Azpurua J, Feigenson M, Bozzella M, Mao Z, Catania KC, Gorbunova V (2009). Hypersensitivity to contact inhibition provides a clue to cancer resistance of naked mole-rat. Proc Natl Acad Sci U S A..

[41] Kowalczyk A, Partha R, Clark NL, Chikina M (2020). Pan-mammalian analysis of molecular constraints underlying extended lifespan. Elife..

[42] Kosova AA, Kutuzov MM, Evdokimov AN, Ilina ES, Belousova EA, Romanenko SA (2019). Poly(ADP-ribosyl)ation and DNA repair synthesis in the extracts of naked mole rat, mouse, and human cells. Aging (Albany NY).

[43] Ray Chaudhuri A, Nussenzweig A (2017). The multifaceted roles of PARP1 in DNA repair and chromatin remodelling. Nat Rev Mol Cell Biol..

[44] Zhao Y, Tyshkovskiy A, Muñoz-Espín D, Tian X, Serrano M, de Magalhaes JP, Nevo E, Gladyshev VN, Seluanov A, Gorbunova V (2018). Naked mole rats can undergo developmental, oncogene-induced and DNA damage-induced cellular senescence. Proc Natl Acad Sci..

[45] Abegglen LM, Caulin AF, Chan A, Lee K, Robinson R, Campbell MS, Kiso WK, Schmitt DL, Waddell PJ, Bhaskara S, Jensen ST, Maley CC, Schiffman JD (2015). Potential mechanisms for cancer resistance in elephants and comparative cellular response to DNA damage in Humans. Jama..

