# Supplementary material for: Isolation and characterization of neural stem/progenitor cells in the subventricular zone of the naked mole-rat brain
Source: Inflamm Regen. 2021 Nov 1;41:31. doi: 10.1186/s41232-021-00182-7 (PMC8559411; doi:10.1186/s41232-021-00182-7)
Supplement: Supplementary file 2 — Additional file 2: Supplementary Figure 1. Immunohistochemical analysis of the neonatal naked mole-rat (NMR) subventricular zone (SVZ). a Immunofluorescence images of nestin (NES) and musashi RNA-binding protein 1 (MSI1) in the neonatal NMR SVZ. The inset shows an overview of Hoechst staining of a coronal hemisection of the neonatal NMR brain. The boxed region in the inset was enlarged. Scale bars: 100 μm (main image) and 500 μm (inset). LV, lateral ventricle. b Immunofluorescence images of BrdU (red) and Hoechst 33342 (blue) in the neonatal NMR SVZ. The boxed regions in the left image are enlarged in the center and right images. Scale bars: 500 μm (left), 100 μm (center), and 100 μm (right). LV, lateral ventricle. c Immunofluorescence image of SRY-box transcription factor 2 (SOX2) in the neonatal NMR SVZ. Scale bar: 100 μm. LV, lateral ventricle. d Multiple alignment of the antigen sites recognized by anti-NES antibodies in the human, NMR, and mouse proteins. [file 41232_2021_182_MOESM2_ESM.pdf]

Supplementary Figure 1

a

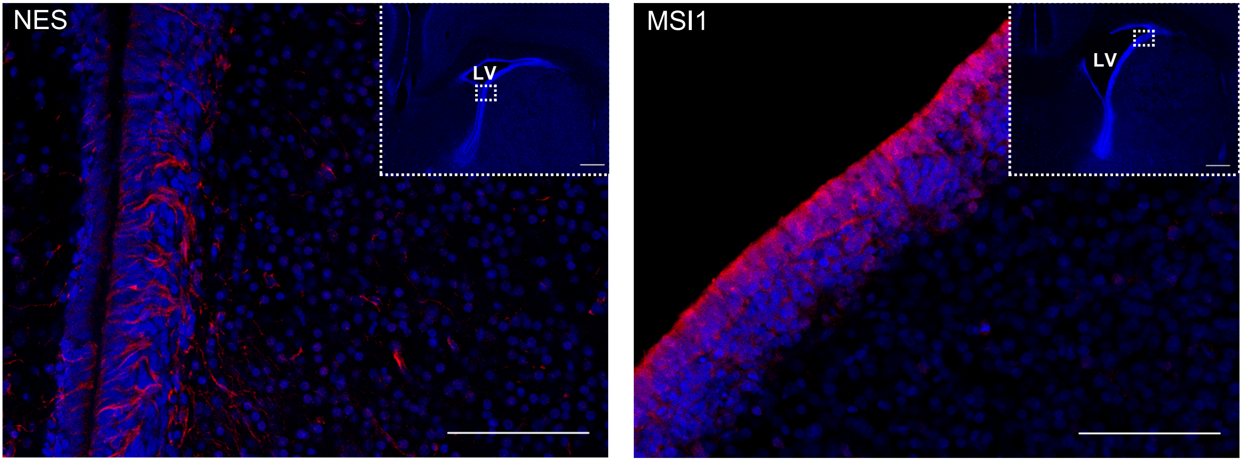

b

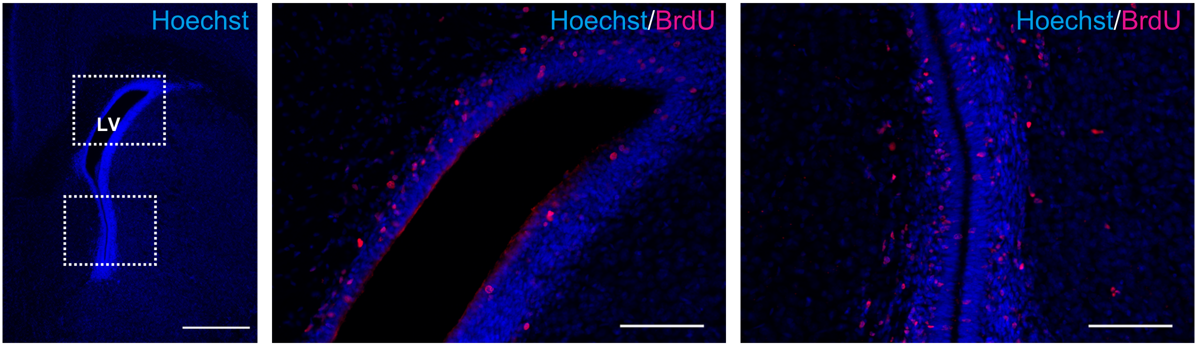

c

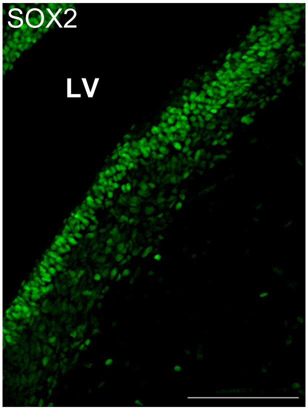

d

		Homology to the epitope
Human (epitope)	VHLGQGQFLKFTQRE	
NMR	LHLGPSQFLKFTPRE	73.3%
Mouse	VHLGPSQPLKFTLSG	60.0%
