# Supplementary figures and images for: Isolation and characterization of neural stem/progenitor cells in the subventricular zone of the naked mole-rat brain
Source: Inflamm Regen. 2021 Nov 1;41:31. doi: 10.1186/s41232-021-00182-7 (PMC8559411; doi:10.1186/s41232-021-00182-7)

## Supplementary Figure 2

a

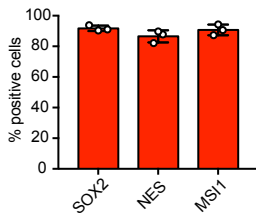

b

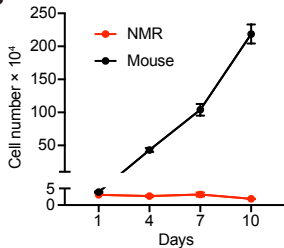

c

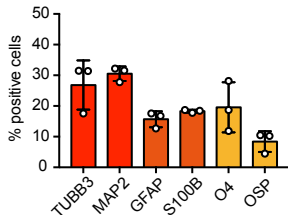

Supplement: Supplementary file 3 — Additional file 3: Supplementary Figure 2. Quantification of the percentages of cells positive for each cell type-specific marker and comparison of the growth of neural stem/progenitor cells (NS/PCs) from naked mole-rats (NMR) and mouse in standard culture condition for mouse-NS/PCs. a Proportions of cells positive for each neural stem cell marker in adherent culture conditions. Data are mean ± standard deviation (SD) from n = 3 biological replicates. b Growth of NMR-NS/PCs and mouse-NS/PCs in adherent culture conditions at 37°C and in 21% O2. Data are mean ± SD of n = 3 biological triplicates. c Proportions of cells positive for each cell type-specific marker in adherent culture conditions. Data are mean ± SD from n = 3 biological replicates. [file 41232_2021_182_MOESM3_ESM.pdf]

## Supplementary Figure 3

**a**

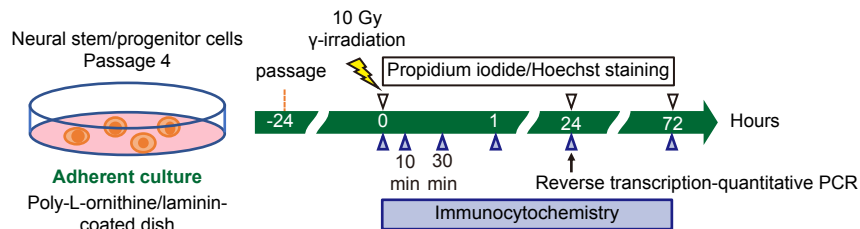

**b**

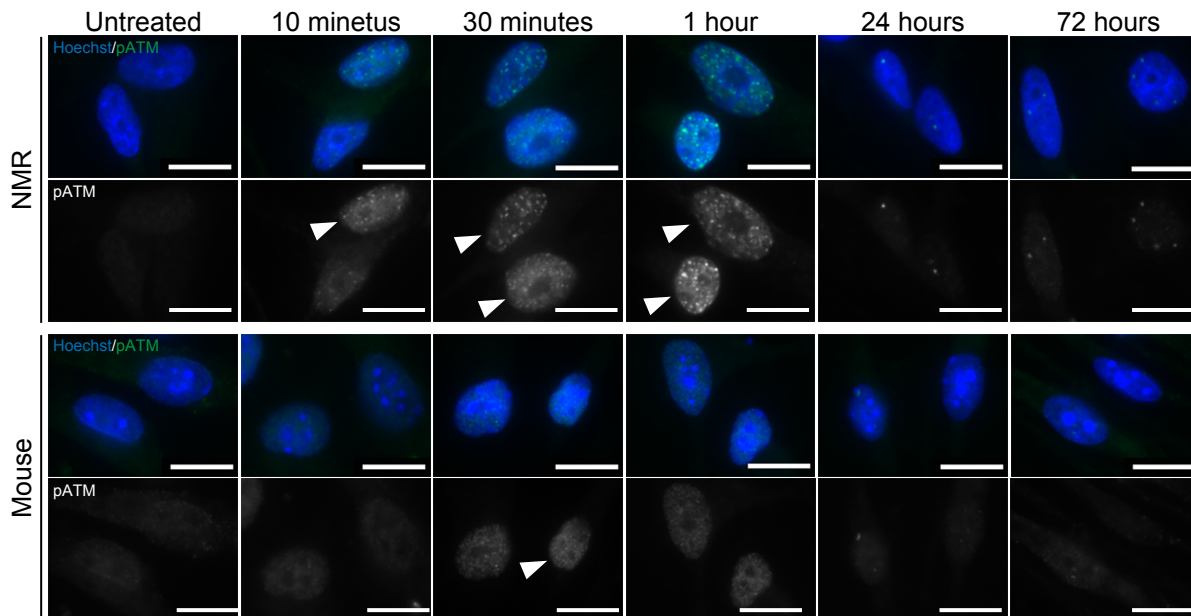

Supplement: Supplementary file 4 — Additional file 4: Supplementary Figure 3. Analysis of the DNA damage response after γ-irradiation. a Timeline of the experimental procedure for γ-irradiation. b Immunofluorescence images of phosphorylated ATM serine/threonine (pATM) in naked mole-rat (NMR) and mouse neural stem/progenitor cells exposed to γ-radiation or left untreated. Blue, Hoechst 33342. White arrowheads indicate positive cells with >10 pATM foci per nuclei. Scale bars: 10 μm. [file 41232_2021_182_MOESM4_ESM.pdf]
